# Impact of early integrated rehabilitation on fatigue in 600 patients with breast cancer – a prospective study

**DOI:** 10.2478/raon-2024-0016

**Published:** 2024-03-07

**Authors:** Masa Auprih, Tina Zagar, Nina Kovacevic, Andreja Cirila Skufca Smrdel, Nikola Besic, Vesna Homar

**Affiliations:** Department of Surgical Oncology, Institute of Oncology, Ljubljana, Slovenia; Slovenian Cancer Registry, Institute of Oncology Ljubljana, Ljubljana, Slovenia; Department of Gynaecological Oncology, Institute of Oncology Ljubljana, Ljubljana, Slovenia; Faculty of Medicine Ljubljana, Ljubljana, Slovenia; Department of Psycho-Oncology, Institute of Oncology Ljubljana, Ljubljana, Slovenia

**Keywords:** early integrated rehabilitation, fatigue, breast cancer, EORTC questionnaire

## Abstract

**Background:**

Fatigue after breast cancer treatment is a common burden that is challenging to treat. The aim of this study was to explore if such integrated rehabilitation program reduces the prevalence of chronic fatigue compared to simple, non-integrated rehabilitation.

**Patients and methods:**

The subjects of our prospective study were 600 female breast cancer patients (29–65 [mean 52 years] of age), who participated in the pilot study on the individualized integrated rehabilitation of breast cancer patients in 2019–2021 and were monitored for one year. The control group included 301 patients and the intervention group numbered 299 patients. The patients completed three questionnaires (EORTC QLQ-C30, -BR23 and NCCN): before cancer treatment, and then six and twelve months after the beginning of cancer treatment. The control group obtained the standard rehabilitation program, while the intervention group was part of the early, individualized multidisciplinary and integrated approach of rehabilitation. The rehabilitation coordinator referred patients for additional interventions (*e.g.*, psychologist, gynecologist, pain management team, physiotherapy, clinical nutrition team, kinesiologist-guided online training, vocational rehabilitation, general practitioner). Data on the patients’ demographics, disease extent, cancer treatment and complaints reported in questionnaires were collected and analyzed.

**Results:**

There were no differences between the control and the intervention group of patients in terms of age, education, disease extent, surgical procedures, systemic cancer treatment, or radiotherapy, and also no differences in the fatigue before the beginning of treatment. However, patients from the control group had a greater level of constant fatigue than patients from the intervention group half a year (p = 0.018) and a year (p = 0.001) after the beginning of treatment. Furthermore, a greater proportion of patients from the control group experienced significant interference with their usual activities from fatigue than from the intervention group, half a year (p = 0.042) and a year (p = 0.001) after the beginning of treatment. A multivariate logistic regression showed that one year after the beginning of treatment, the only independent factor correlated to fatigue was inclusion into the intervention group (p = 0.044). Inclusion in the intervention group was beneficial—patients from the control group were 1.5 times more likely to be fatigued.

**Conclusions:**

Early individualized integrated rehabilitation is associated with a lower prevalence of chronic fatigue or fatigue interfering with usual activities in breast cancer patients in comparison to the control group of patients.

## Introduction

Breast cancer is the most common malignancy in women worldwide.^1^ New diagnostic options and treatments result in a survival rate as high as 85–90% after five years in developed countries which sets a new challenge for health care systems – how to successfully improve the quality of life of breast cancer patients during and after the treatment.^2,3,4,5^ The most important tool in achieving a good quality of life is early, optimized, individualized and integrated rehabilitation adapted to the needs of each patient.^2^

Fatigue is one of a number of burdens for breast cancer patients, which is caused by the cancer itself or its treatment. Fatigue is characterized by persistent physical, emotional, and cognitive tiredness related to cancer and/or cancer treatment that is not proportional to recent physical activity, interferes with usual functioning and is not relieved by rest or sleep.^6,7^ It can also be a barrier to cancer survivors’ return to work.^8^ A meta-analysis, which included 12,327 breast cancer survivors, reported that approximately one in four breast cancer survivors suffer from severe fatigue.^9^ Fatigue usually improves after the treatment, but it can also have long-term effects and can progress to chronic fatigue.^9^

Rehabilitation can help persons with chronic disease or impairment to achieve and maintain the highest possible physical, social, psychological, and occupational functioning.^10^ Rehabilitation is a dynamic process that starts with the diagnosis and continues to the end of life. Implementation of guidelines for fatigue evaluation and management is best accomplished by an interdisciplinary team who are able to tailor interventions to the needs of the individual patient.^11^ Patients are therefore referred to an appropriate health care provider − survivorship, palliative care, integrative oncology, psychology, psychiatry, physical therapy, vocational therapy, and/or physical medicine. The results are best if rehabilitation starts early, ideally before the beginning of the treatment.^11^

Breast cancer patients in this study were offered an improved rehabilitation program, that started soon after diagnosis and was tailored to individual needs. The aim of this study was to explore if such integrated rehabilitation program reduces the prevalence of chronic fatigue compared to simple, non-integrated rehabilitation.

## Patients and methods

### Patients

**FIGURE 1.**
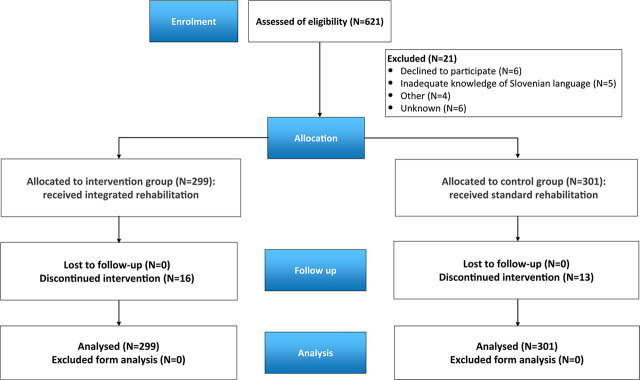
A flowchart of patients’ inclusion in our study.

A prospective pilot study included patients that were diagnosed and treated at the Institute of Oncology Ljubljana (IOL), Slovenia, from 2019 to 2022. Consecutively, 600 patients with all stages of invasive breast cancer and aged less than 65 years at the time of diagnosis were included. The exclusion criterion was if the patient refused to participate in the study or was unable to fill in the questionnaires. A flowchart of patients’ inclusion in our study is presented in Figure 1. The study was reviewed and approved by the Protocol Review Board (ERID-KSOPKR-0086/2019) and the Ethics Committee of the Institute of Oncology Ljubljana (ERIDEK-0102/2019). The study was performed in accordance with the ethical standards laid down in the appropriate version of the 1964 Declaration of Helsinki and conducted with the understanding and consent of all the subjects involved.

All 600 planned patients were included in the study by December 2021. The control group consisted of 301 patients that were included in the study from December 2019 to the end of March 2021 and had already received existing routine non-integrated rehabilitation, *i.e.*, without a systematic patient needs evaluation and preemptive measures. Implementation of non-integrated rehabilitation began only if the individual patient specifically highlighted her problem in the outpatient clinic and/or when the attending physician noticed the need of the individual patient and directed her to appropriate treatment.

The inclusion of 299 patients in the intervention group started in September 2020 and ended in December 2021. In the intervention group, we included only those patients who live near the OIL, as we wanted them to be able to come twice a week to exercise in Ljubljana. The patients in the intervention group received integrated and individualized rehabilitation accordingly to the IOL’s clinical guidelines and pathway of integrated rehabilitation developed specially for this study. Due to the COVID-19 pandemic, the study was prolonged with respect to the initial timeframe to reach the targeted number of participants.

### Study protocol

During scheduled check-ups with the oncologist, each patient answered three standardized questionnaires (the European Organization for Research and Treatment of Cancer [EORTC] QLQ-C30, -BR23 and the National Comprehensive Cancer Network [NCCN]) before the treatment, half a year, and one year after the beginning of the treatment. The EORTC quality of life questionnaires (QLQ) and NCCN questionnaire are an integrated system for assessing the health-related quality of life of cancer patients.^11,12,13^ The EORTC QLQ-C30 consists of a global health quality of life scale, five functional scales (physical, role, emotional, cognitive, and social function), and symptom scales (fatigue, nausea and vomiting, pain, dyspnea, insomnia, appetite loss, constipation, diarrhea, and financial difficulties).^12^ The EORTC QLQ-BR23 consists of symptom scales of systemic therapy side effects (upset by hair loss, arm symptoms, breast symptoms) and functional scales (body image, future perspective, sexual functioning, and sexual enjoyment).^13^ The NCCN questionnaire included questions about cardiac health, anxiety, depression, distress, cognitive function, fatigue, lymphedema, pain, hormone-related symptoms, sexual function, sleep disorder, healthy lifestyle (regular physical activity or exercise, diet, weight, use of vitamins or other supplements, smoking and consumption of alcohol), employment, and return to work.^11^

After completing all three standardized questionnaires, each patient also had an interview with a specialized registered nurse—a rehabilitation coordinator. The coordinator recorded the patient’s most important needs and specific circumstances. The documentation of each patient from the intervention group was discussed at the multidisciplinary meeting for integrated rehabilitation before, half a year, and one year after the beginning of treatment. The multidisciplinary team consisted of an integrative rehabilitation coordinator, surgical oncologist, radiation oncologist, medical oncologist, psychologist, psychiatrist, general practitioner, physiotherapist, psychiatrist, specialist in medical rehabilitation and physical medicine, specialist in vocational medicine, and gynecologist.^14^ The aim was to identify the patient’s problems early, predict the late treatment consequences, implement measures to prevent or diminish the patient’s problems and start rehabilitation as soon as needed. The mainstay of the patient’s integrative rehabilitation was educating and empowering the patient to self-care and to be able to manage her symptoms and prevent undesired side effects of treatment, as already described in our recent publication.^14^

### Management of a patient with fatigue

All patients were screened for fatigue with questionnaires as recommended in the NCCN clinical practice guidelines for survivorship, IOL’s guidelines and clinical pathway of integrated rehabilitation.^11,15,16^ The fatigue was graded in four grades (1 - without, 2 - mild, 3 - moderate, or 4 - severe) according to the EORTC QLQ-C30 questionnaire.^12^ According to the NCCN questionnaire^11,15,16^ the level of fatigue was assessed with a quantitative or semi-quantitative assessment on a 0 to 10 numeric rating scale (zero = no fatigue and 10 = worst fatigue imaginable). Mild fatigue had a score of 1 to 3, moderate fatigue 4 to 6, and severe fatigue 7 to 10.

According to the IOL guidelines and clinical pathway, the individualized integrated rehabilitation was carried out on three levels.^15,16^ The first level was the treatment of all diseases and conditions that contribute to fatigue or may cause an increased baseline level of fatigue. The patients with moderate to severe fatigue (numeric scale from 4 to 10) were evaluated by the oncologist and/or general practitioner with regard to current disease status, history and physical examination, review of current medications, review of organ systems, and evaluation of other concurrent symptoms and contributing factors. The most important diseases that affect baseline fatigue such as heart failure or chronic kidney disease, thyroid malfunction, and/or anemia were ruled out clinically and by laboratory tests.

Secondly, all our patients from the control and intervention group were educated about a healthy lifestyle and were offered various techniques and training to help them cope with fatigue. All patients received written information about these topics and had information available on the website of the IOL dedicated to integrative rehabilitation. Prevention of fatigue is especially important before starting chemotherapy. Education and counseling are believed to be central to the effective management of fatigue^11^ and the rehabilitation coordinator devoted a lot of time to patient education during each patient’s visit. Cancer patients were encouraged to engage in regular moderate physical activity for at least 150 minutes per week and were educated about appropriate exercise to reduce fatigue. Patients were advised to be physically active each day by walking, cycling, doing resistance exercise, or a combination of aerobic and resistance exercise. All our patients from the intervention group who were treated with chemotherapy or reported fatigue had been asked to join a physical activity guided by a kinesiologist twice a week conducted online by a videoconference. On average more than 30 patients attended each videoconference. Advice on maintaining a healthy diet was given during a visit to the Clinical Nutrition and Dietotherapy outpatient clinic at our Institute as well as during online workshops guided by experienced clinical nutritionists. Since November 2021, the patients with fatigue from the intervention group were recommended to join the videoconferences with a yoga teacher once a week.

Thirdly, patients from the intervention group with moderate or severe fatigue were referred for consultations and treatment of fatigue to the oncologist, general practitioner, clinical psychologist or psychiatrist, pain relief clinic acupuncture, and/or yoga. All the interventions were covered by health insurance. Psychosocial interventions were recommended to all our patients with moderate to severe fatigue. These were available sooner for the first half of the intervention group than for the control group of patients as the COVID-19 pandemic prevented group therapies from taking place and enabled individual therapies from March 2020 onwards. However, because of a shortage of clinical psychologists in our country it was more difficult to obtain psychosocial intervention for the second part of the intervention group of patients. IOL’s psycho-oncology department provided psychological counseling, crisis interventions, and cognitive behavioral psychotherapy. Evaluation at the psycho-oncology department was done during the first year after the beginning of oncological therapy in the intervention and control group of patients in 127 and 42 patients, respectively. Altogether 36 patients from the intervention group attended from one to eight (median 5.6) online group meetings with a clinical psychologist.

Depending on the patient’s needs, the patient was referred also to other healthcare providers within the framework of the Slovenian health system. Anesthesiologists from the Institute of Oncology Ljubljana offered acupuncture as well as pharmacological therapy. General practitioners had the possibility to refer the patient to a number of workshops held at the Center for Health Promotion, which operates within the community health centers.

### Statistical analysis

Data on the patients’ demographics, disease extent, cancer treatment, fatigue, and other complaints reported in questionnaires were collected and managed in REDCap (Research Electronic Data Capture) Version 12.4.22. Additional data processing was performed in Excel (Microsoft Office Professional Plus 2016). The average score of all answers to questions from EORTC questionnaires about different function scales and symptoms was standardized with a linear transformation on a scale from 0 to 100. Differences between scores between the intervention and control groups at the same time point were assessed with the Wilcoxon signed-ranks test. Differences measured at two time points (in the same persons) used the Wilcoxon signed-rank paired difference test. Distribution between categories was analyzed using the chi-square test. ANOVA was used to test for differences in the means of three or more groups. Differences between the answers from the intervention and control groups to questions from NCCN questionnaires at the same time point were assessed with the ANOVA test. All statistical analyses were done in Version 27 of the SPSS Statistical Software and Software R version 4.2.2. P-values under 0.05 were considered statistically significant.

## Results

Data about patients, disease characteristics, and treatment are presented in Table 1. There were no differences between the control and the intervention group of patients in terms of age, education, disease extent, surgical procedures, systemic cancer treatment, or radiotherapy. As expected, both groups of patients differed in living areas. The majority of patients from the intervention group lived in urban areas, while patients from the control group were more distributed between rural and suburban areas. Namely, it was planned that the patients from the intervention group would exercise under the supervision of a kinesiologist in the gym close to our Institute, so only the patients from central Slovenia were included in the intervention group. However, due to the COVID-19 pandemic, we could not do physical exercise in the gym, so it was done online instead.

**TABLE 1.** Demographic and clinical characteristics of patients, pathological characteristics of tumors and treatment. P-value refers to difference between control and intervention group; it is calculated by t-test in case of comparing means and by chi-squared test in case of counts

**Factor**	**Subgroup**	**All patients (N = 600)**	**Control group (N = 301)**	**Intervention group (N = 299)**	**P-value**
**Mean age of patients (years)**		50.78	50.59	50.97	0.601
**Living areas**	Urban	287	125	162	0.003
	Suburban	105	53	52	
	Rural	208	123	85	
**Education (N = 599)**	Primary school	66	39	27	0.290
	Secondary school	242	117	125	
	Higher	291	144	147	
**Socioeconomic status**	Low	71	36	35	0.940
	Middle	432	217	215	
	Higher	95	46	49	
**With whom they live (N = 597)**	Alone	58	24	34	0.600
	With partner only	145	71	74	
	Partner and children	289	147	142	
	With children only	42	22	20	
	Other	63	35	28	
**Employment (N = 581)**	Unemployed	54	35	19	0.067
	Employed	433	209	224	
	Retired	94	45	49	
**Mean primary tumor size (mm)**		26.3	25.5	27.2	0.285
**Tumor stage**	In situ	10	5	5	0.152
	I	260	133	127	
	II	214	97	117	
	III	81	50	31	
	IV	35	16	19	
**Concomitant diseases**	No	301	154	147	0.624
	Yes	299	147	152	
**Neoadjuvant chemotherapy and/or anti-HER-2 therapy**	No	465	227	237	0.241
	Yes	135	74	61	
**Breast surgery**	Mastectomy	252	135	117	0.357
	Tumorectomy	326	156	170	
	No surgery	22	10	12	
**Lymph node surgery**	Lymphadenectomy	151	83	86	0.337
	Sentinel node biopsy	417	204	213	
	No surgery	32	14	18	
**Breast reconstruction**	No	431	214	217	0.402
	Tissue expander	127	69	58	
	Free-flap	42	18	24	
**Breast external beam radiotherapy**	No	149	83	66	0.131
	Yes	451	218	233	
**Chemotherapy**	No	280	137	143	0.623
	Yes	320	164	156	
**Anti-HER2 therapy**	No	522	264	258	0.629
	Yes	78	37	41	
**Hormone therapy**	No	132	69	63	0.623
	Yes	468	232	236	

### EORTC questionnaires

**FIGURE 2.**
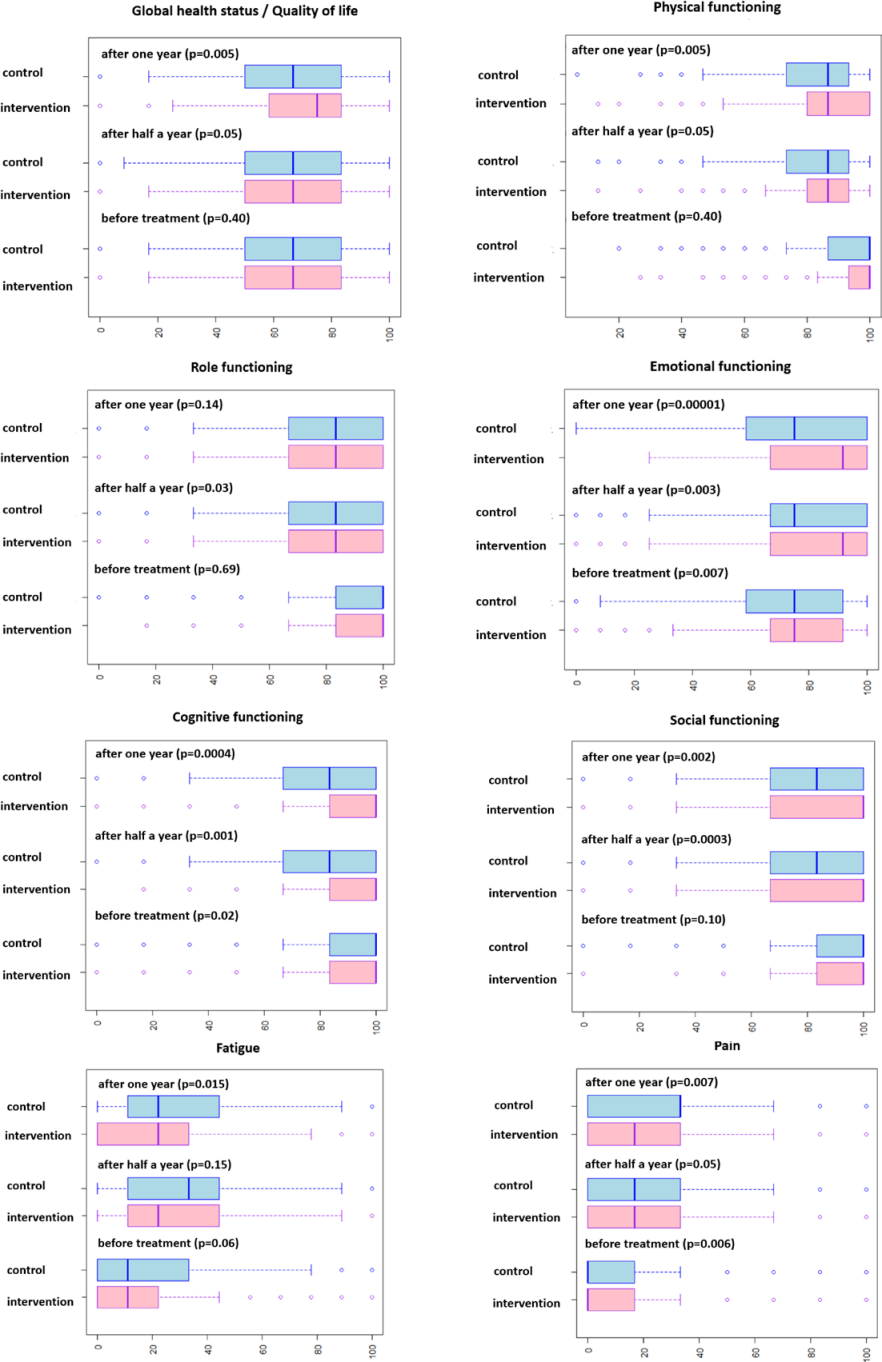
Data and statistical analysis from EORTC C30 questionnaires about the global health quality of life scale, physical, role, emotional, cognitive, and social function scale, symptom scales about fatigue, pain and insomnia in the intervention and control group of patients before treatment, half a year, and a year after the beginning of treatment.

Data and statistical analysis from EORTC C30 questionnaires about the global health quality of life scale, physical, role, emotional, cognitive, and social function scale, symptom scales about fatigue, pain, and insomnia in the intervention and control group of patients before treatment, half a year, and a year after the beginning of treatment are presented in Figure 2. Before the treatment, the patients from the intervention group reported significantly fewer problems in emotional and cognitive function scale and pain in comparison to the control group. Half a year after the beginning of treatment, the patients from the intervention group reported significantly fewer problems on the physical, role, emotional, cognitive, and social function scale, and pain in comparison to the control group. A year after the beginning of treatment, the patients from the intervention group reported significantly fewer problems on the global health quality of life scale, physical, emotional, cognitive, and social function scale, fatigue, and pain in comparison to the control group.

**FIGURE 3.**
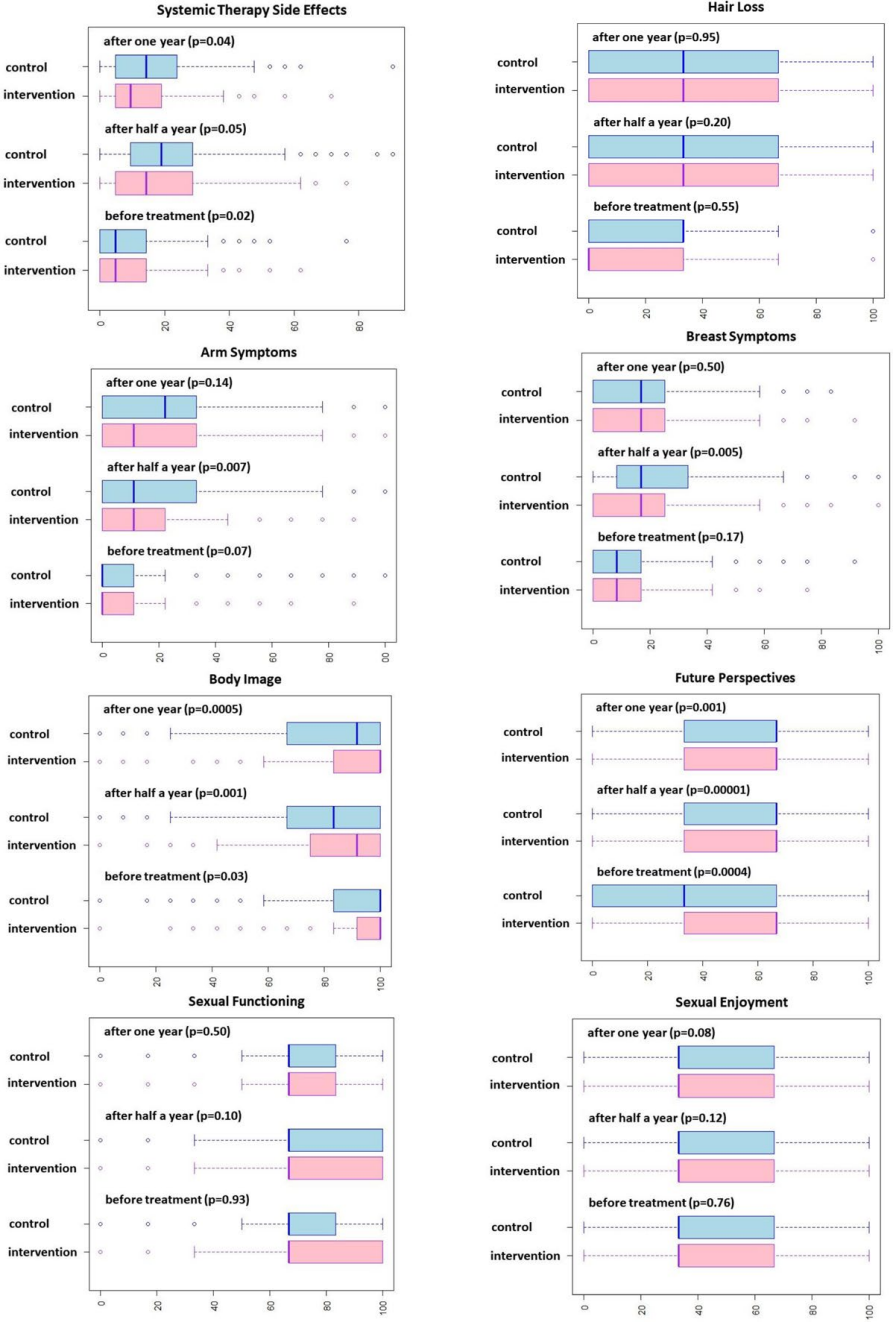
Data and statistical analysis from EORTC BR23 questionnaires.

Data and statistical analysis from EORTC BR23 questionnaires are presented in Figure 3. Before the treatment, the patients from the intervention group reported significantly fewer problems with systemic therapy side effects but were more concerned about body image and future perspectives in comparison to the control group. Half a year after the beginning of treatment, the patients from the intervention group reported significantly fewer problems with systemic therapy side effects, arm symptoms, and breast symptoms, but were still more concerned about body image and future perspectives in comparison to the control group. A year after the beginning of treatment, the patients from the intervention group reported significantly fewer problems with systemic therapy side effects but were still more concerned about body image and future perspectives in comparison to the control group.

### NCCN questionnaires

Table 2 shows mean values of psychological factors and pain reported by patients and assessed on a 0 to 10 numeric rating scale (zero = no pain and 10 = worst imaginable) before, half a year, and one year after the beginning of treatment. Before the treatment, the patients from the intervention group reported a significantly lower level of depression, anxiety, and pain in comparison to the control group. Half a year and one year after the beginning of treatment, the patients from the intervention group reported a significantly lower level of depression, anxiety, difficulty concentrating, disturbing fatigue, insomnia, and pain in comparison to the control group.

**TABLE 2.** Mean values of psychological factors and pain reported by patients before, half a year and one year after the beginning of treatment

**Factor**	**Time of assessment**	**Group**	**Mean value**	**Standard deviation**	**p-value**
**Depression level**	Before therapy	Control	4.2	2.8	0.013
		Intervention	3.6	2.5	
	After half year	Control	3.0	2.4	< 0.001
		Intervention	2.2	1.7	
	After one year	Control	3.2	2.4	< 0.001
		Intervention	2.3	1.9	
**Anxiety level**	Before treatment	Control	4.2	2.7	0.041
		Intervention	3.8	2.6	
	After half year	Control	3.2	2.5	< 0.001
		Intervention	2.4	1.8	
	After one year	Control	3.5	2.6	< 0.001
		Intervention	2.6	2.0	
**Level of difficulty concentrating**	Before treatment	Control	3.2	2.5	0.96
		Intervention	3.1	2.3	
	After half year	Control	3.2	2.4	0.02
		Intervention	2.8	2.1	
	After one year	Control	3.6	2.4	< 0.001
		Intervention	2.7	2.1	
**Constant fatigue**	Before treatment	Control	3.2	2.4	0.59
		Intervention	3.1	2.2	
	After half year	Control	3.8	2.6	0.018
		Intervention	3.3	2.4	
	After one year	Control	4.0	2.7	0.001
		Intervention	3.3	2.3	
**Disturbing fatigue**	Before treatment	Control	2.8	2.3	0.50
		Intervention	2.9	2.2	
	After half year	Control	3.9	2.5	0.003
		Intervention	3.3	2.3	
	After one year	Control	3.8	2.5	< 0.001
		Intervention	3.2	2.2	
**Insomnia**	Before treatment	Control	4.2	3.0	0.22
		Intervention	3.9	2.8	
	After half year	Control	4.8	3.0	0.002
		Intervention	4.0	2.7	
	After one year	Control	4.8	3.0	< 0.001
		Intervention	3.9	2.9	
**Pain**	Before treatment	Control	2.8	2.4	0.005
		Intervention	2.3	2.0	
	After half year	Control	3.4	2.4	0.006
		Intervention	2.9	2.1	
	After one year	Control	3.7	2.5	< 0.001
		Intervention	2.7	1.8	

Regarding the proportion of patients with physical activity of at least 150 minutes per week, there was no difference between the groups before treatment (p = 0.73), but after one year the difference was statistically significant (p = 0.034). Before the cancer treatment, smoking was present in the intervention and control group in 22% and 27% (p = 0.27), respectively. However, one year after the beginning of cancer treatment, smoking was less common in the intervention group in comparison to the control group of patients (p = 0.001).

### Fatigue

Regarding question 18 from the EORTC C30 questionnaire, 50% of the patients answered that they were not tired when asked before the beginning of the treatment. After half a year and one year after the beginning of treatment the answer was no in only 32% and 34%, respectively.

**FIGURE 4.**
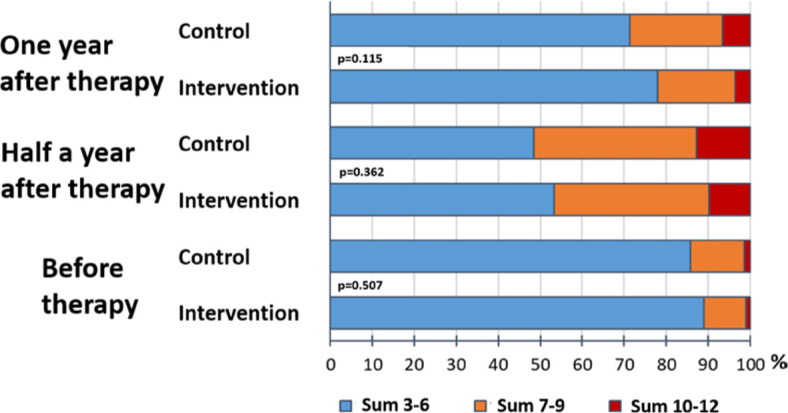
The sum of responses to EORTC questions 10, 12 and 18 in the intervention and control groups before, half a year, and a year after treatment.

The symptom of fatigue was assessed with the sum of EORTC questions number 10, 12, and 18 on the Likert scale (1–without, 2–mild, 3–moderate, 4–severe). The sum of all three answers to EORTC questions before, after half a year, and after a year after treatment can be a minimum of 3 and a maximum of 12. Figure 4 shows the sum of responses to EORTC questions 10, 12, and 18 in the intervention and control groups before, half a year, and a year after treatment. We considered that the patient has moderate or severe fatigue when the sum of all three responses was equal to seven or more or at least one of the patient’s responses was “4–severe”. Fatigue was present in all our patients before treatment, half a year, and a year after treatment in 12.7%, 47.7%, and 24.2%, respectively.

The univariate association of each individual variable on fatigue by individual logistic regressions and multivariate models’ logistic regression about the association of all included variables simultaneously and fatigue are presented in Table 3 and Table 4, respectively.

**TABLE 3.** The influence of each individual variable on fatigue (univariate models) by individual logistic regressions before treatment, half a year and one year after the beginning of treatment. OR – odds ratio; CI – confidence interval

**Before treatment**	**OR**	**95% CI**	**p-value**
Control group	1.3	0.8–2.2	0.245
**Half a year after the beginning of treatment**	**OR**	**95% CI**	**p-value**
Control group	1.3	0.9–1.9	0.114
Age group 45–54 years	1.0	0.7–1.6	0.855
Age group 55–64 years	0.9	0.6–1.5	0.803
Chemotherapy – yes	1.3	0.9–1.9	0.135
Hormonal therapy – yes	0.9	0.6–1.4	0.689
Radiotherapy – yes	0.8	0.5–1.3	0.364
Neoadjuvant chemotherapy and/or anti-HER2 therapy	1.3	0.9–1.9	0.227
Surgery – not done	1.7	0.6–4.4	0.308
Surgery – Tumorectomy – yes	1.3	0.9–1.8	0.208
Axillary lymphadenectomy – yes	0.7	0.5–1.1	0.162
Breast reconstruction – yes	0.8	0.5–1.2	0.256
Presence of distant metastases	1.4	0.7–2.8	0.403
**A year after the beginning of treatment**	**OR**	**95% CI**	**p-value**
Control group	1.5	1.0–2.2	**0.046**
Age group 45–54 years	1.2	0.8–2.0	0.387
Age group 55–64 years	0.6	0.4–1.0	0.064
Chemotherapy – yes	1.4	0.8–1.7	0.493
Hormonal therapy – yes	1.3	0.8–2.2	0.246
Radiotherapy – yes	0.7	0.5–1.2	0.191
Neoadjuvant chemotherapy and/or anti-HER2 therapy	1.2	0.8–1.9	0.347
Surgery – not done	1.7	0.5–5.2	0.371
Surgery – Tumorectomy – yes	1.1	0.7–1.6	0.961
Axillary lymphadenectomy – yes	0.7	0.5–1.1	0.161
Breast reconstruction – yes	0.9	0.6–1.3	0.454
Presence of distant metastases	1.8	0.8–4.0	0.130

**TABLE 4.** The influence of all included variables simultaneously on fatigue half a year and a year after the beginning of treatment

	**Half a year after treatment**			**One year after treatment**		

	**OR**	**95% CI**	**p-value**	**OR**	**95% CI**	**p-value**
Control group	1.4	0.9–2.0	0.100	1.5	1.0–2.2	**0.044**
Age group 45–54 years	1.1	0.7–1.7	0.822	1.1	0.7–1.9	0.617
Age group 55–64 years	1.0	0.6–1.6	0.931	0.6	0.3–1.0	0.052
Chemotherapy – yes	1.6	1.1–2.5	**0.025**	1.3	0.9–2.1	0.206
Hormonal therapy – yes	1.1	0.7–1.8	0.616	1.6	0.9–2.6	0.088
Radiotherapy – yes	0.7	0.4–1.2	0.196	0.7	0.4–1.3	0.277
Surgery – not done	1.2	0.3–4.4	0.745	0.9	0.2–3.9	0.879
Surgery – Tumorectomy – yes	1.6	0.9–2.6	0.080	1.4	0.8–2.4	0.256
Axillary lymphadenectomy – yes	0.7	0.4–1.2	0.232	0.7	0.4–1.2	0.148
Presence of distant metastases	1.4	0.5–3.7	0.491	2.1	0.8–5.9	0.145

OR = odds ratio; CI = confidence interval

A multivariate logistic regression analysis showed that half a year after the beginning of treatment, fatigue was only associated with treatment with chemotherapy. Patients who received chemotherapy were 1.6 times more likely to be fatigued in comparison to those without chemotherapy. But, one year after the beginning of treatment, treatment with chemotherapy was no longer associated with fatigue. The only independent factor correlated to fatigue was inclusion into the intervention group. Inclusion into the intervention group was beneficial; patients from the control group were 1.5 times more likely to be fatigued.

Answers to the NCCN questionnaires show that there were no differences between the groups regarding constant fatigue before treatment (p = 0.59). However, patients from the control group had a greater level of fatigue than patients from the intervention group half a year (p = 0.018) and a year (p = 0.001) after the beginning of treatment. Furthermore, there were no differences in mean value between both groups regarding fatigue interfering with usual activities before therapy (0.50). Patients from the control group had more fatigue interfering with usual activities than from the intervention group half a year (p = 0.003) and a year (p < 0.001) after the beginning of treatment.

## Discussion

Most published reports on oncological rehabilitation include patients who started rehabilitation after oncological treatment and a minority of reports focus on rehabilitation during oncological treatment. The purpose of our study was to improve the rehabilitation of our patients with breast cancer and to start implementing integrated rehabilitation. Breast cancer patients in this study were offered an improved rehabilitation program that started early after diagnosis and was tailored to individual needs. The aim of this study was to explore if such integrated rehabilitation program reduces the prevalence of chronic fatigue compared to simple, non-integrated rehabilitation. We expected that earlier rehabilitation would reduce the patients’ difficulties and side effects of treatment, so our patients from the intervention group started with integrative oncological rehabilitation already at the beginning and it was carried on also during oncological treatment. Our results show that patients who received integrated rehabilitation reported significantly less fatigue and better quality of life compared to controls.

Before treatment, our two groups of patients did not differ in terms of fatigue, as the two groups did not differ in terms of risk factors for fatigue (age, education level, stage of disease, and extent of treatment). This is understandable, since we allocated the vast majority of patients to the two groups almost randomly according to the time of treatment; in one group there were patients who started treatment before the other group of patients. The essential reason for lower fatigue in the intervention group is that these patients received a number of measures that have been proven to reduce fatigue. The mainstay of our integrative rehabilitation was patient education about what they themselves can do to manage their symptoms, and to mitigate or even prevent the adverse effects of treatment. In contrast to the control group, the patients from the intervention group had 3 interviews with the integrated rehabilitation coordinator, who during each interview educated patients about the prevention and treatment of fatigue. All patients were referred to a general practitioner for counseling on leading a healthy lifestyle. Additionally, patients from the intervention group were advised to be physically active and were provided with physical exercise guided by a kinesiologist twice per week, which was carried out online. They also had the possibility to practice yoga. Furthermore, some patients with fatigue had psychotherapy interventions and acupuncture.

Fatigue in breast cancer patients is a common symptom and varies between different phases of breast cancer treatment.^9,17,18^ Reinertsen *et al.*^17^ investigated levels of fatigue in women before, during chemotherapy and at a two-year follow-up. Chronic fatigue was reported before treatment, during chemotherapy and two years after the therapy in 8%, 12%, and 36% of patients, respectively. In our patients, fatigue was present before treatment, half a year, and a year after treatment in 13%, 48%, and 24%, respectively. The degree of difference between our and Norwegian patients regarding fatigue is probably related to the different tests that were used in our and the Norwegian study. Namely, we used EORTC questionnaires, while they also used a specific fatigue questionnaire. A meta-analysis showed that after completion of cancer treatment severe fatigue was present in 22% to 42% of 12,327 breast cancer survivors and that risk factors for chronic fatigue were demographic, the stage of disease, and the extent of oncological therapy.^9^ The relatively low proportion of fatigue reported by our patients one year after the start of treatment (24%), compared to the data from the above-mentioned meta-analysis, could be attributed to the successful measures received by the intervention group of patients, which also reduced the proportion of fatigue in both groups together.

Many studies reviewed by Ruiz-Casado *et al.*^19^ reported that younger and less educated women had greater fatigue. However, a higher level of education was significantly associated with moderate to severe fatigue in patients treated with aromatase inhibitors.^20^ Patients with a partner were less susceptible to severe fatigue than those without a partner.^9^ On the other hand, many patients have problems with fatigue even before starting treatment, and this problem may persist or even worsen during treatment.^18^ Preexisting comorbid conditions or medications used to treat them may contribute to increased fatigue early during cancer treatment.^18,19^ Such conditions include heart disease, hypertension, diabetes, anemia, obesity, arthritis, or psychiatric conditions.^18,19^ Patients who experience psychosocial distress at baseline and patients who have a history of depression are prone to suffer from chronic fatigue.^18^ Our patients from the intervention and control groups did not differ in terms of age, educational structure, or accompanying diseases, so the rate of fatigue between both groups of patients was not different before the beginning of treatment. However, these factors might have contributed to the difference in fatigue rate half a year and one year after the beginning of treatment. At the time of diagnosis, it is impossible to influence any of the studied independent risk factors for chronic fatigue. However, our results clearly show that integrated rehabilitation, although not able to influence individual risk factors, reduces the likelihood of developing chronic or severe fatigue compared to standard care.

Risk of fatigue is significantly higher in patients treated with chemotherapy.^9,18^ Our univariate analysis showed that the patients treated with chemotherapy had increased risk for fatigue half a year and one year after the beginning of treatment. Furthermore, multivariate logistic regression showed that after half a year, fatigue was the only factor associated with treatment with chemotherapy. Patients who received chemotherapy were 1.6 times more likely to be fatigued than those without chemotherapy. This is much more common than the 1.12 times more reported in the meta-analysis by Abrahams *et al.*^9^ However, one year after the beginning of therapy, treatment with chemotherapy was not an independent factor associated with fatigue and inclusion in the intervention or control group of patients was the only independent factor associated with fatigue. We assume that integrated oncological rehabilitation decreased fatigue in patients from the intervention group, while patients from the control group still experienced fatigue.

Conditions that are a consequence of cancer treatment such as insomnia or pain can also contribute to fatigue.^18^ Integrated rehabilitation effectively decreased insomnia and pain in our intervention group of patients. Before treatment, there were no differences in the frequency of insomnia between both groups of patients. However, after half a year and a year, insomnia was more common in the control group of patients than in the intervention group. Furthermore, severe pain in patients from the control group one year after the beginning of treatment was significantly more common than before the treatment. On the other hand, the proportion of patients with severe pain in the intervention group did not significantly change over time.

Several interventions could have positive effects on a specific symptom or a patient’s problems, and timing of the intervention is important.^2^ Proven interventions for prevention or treatment of fatigue are aerobic exercises, resistance training, yoga, psychological interventions (cognitive-behavioral therapy, psychoeducation, mindfulness), healthy lifestyle interventions, acupuncture, and pharmacotherapy.^18^ Our intervention group of patients were advised and received many of these interventions, while the control group of patients did not receive these interventions to the same degree.

Aerobic exercise and resistance training is associated with an important reduction of fatigue in the majority of systematic reviews.^2^ Longer duration, length, and frequency of physical activity has a stronger effect on reducing fatigue.^2^ Furthermore, physical activity reduces fatigue if performed during or after chemotherapy and/or radiotherapy treatments.^21^ Based on these findings, our patients from the intervention group who had chemotherapy or fatigue had been recommended to join a physical activity guided by a kinesiologist twice a week. One year after the beginning of treatment, a significantly larger proportion of patients from the intervention group became more physically active compared to those from the control group. Juvet *et al.*^21^ in a meta-analysis of patients treated with chemotherapy found that fatigue was significantly lower in patients who received physical activity intervention in comparison to controls. In the group with physical activity intervention in comparison to controls during and after oncological treatment they reported a lower standard mean difference of fatigue of 0.19 and 0.52, respectively. Similarly, half a year and a year after the beginning of treatment, the rate of fatigue in our integrated rehabilitation group in comparison to the control group was 1.3 and 1.5 lower, respectively. Furthermore, early integrated rehabilitation helped smoking cessation in a significantly larger proportion of patients from the intervention group compared with the control group^14^, adding to the healthier lifestyle of the patients.

Psychological interventions are the second most effective way to reduce fatigue after physical activity.^22^ Moreover, the combination of physical activity and psychological interventions is even more effective than physical activity or psychological interventions per se.^22^ During oncological treatment, fatigue may be effectively reduced with relaxation exercise, massage, cognitive-behavioral therapy, yoga, and different combinations of these.^23^ Lack of clinical psychologists and psychotherapists in Slovenia makes it difficult to access psychotherapy. Although we had planned that all patients from the intervention group who needed it could receive psychological treatment or psychotherapy, we did not manage to reach this goal. During and after the COVID-19 pandemic access to psychological treatment or psychotherapy treatment became even more difficult than before the pandemic. The COVID-19 pandemic led to a sharp increase in demand for psychological treatment or psychotherapy in the general population. Despite these limitations in access to psychological treatment or psychotherapy, the intervention group had significantly fewer problems compared to the control group in the global health quality of life, physical, role, emotional, cognitive, and social function scale, fatigue, and pain one year after the beginning of treatment.

Another important factor which reduces fatigue is psychoeducation^24,25^ helping participants cope with problems related to breast cancer, teaching stress management strategies, and teaching adaptive strategies improve patients’ quality of life.^26^ A Cochrane review by Bennett *et al.*^27^ provided preliminary findings for the beneficial effect of educational interventions for reducing general cancer-related fatigue, fatigue intensity, fatigue distress, and fatigue interference compared with usual care. Yoga is associated with a significant improvement in quality of life and reduction of fatigue^2,18^; furthermore, acupuncture is effective for the management of fatigue particularly during anti-cancer treatment.^28^ Based on these facts, we tried to include these interventions as much as possible in the rehabilitation of our patients and to provide psychoeducation for our patients. Group and individual behavioral psychotherapy and an individual interview with a psychologist and acupuncture were carried out. Furthermore, the patients from the intervention group attended nutrition workshops and yoga classes online. In addition, all patients had access to online publications about cancer diagnosis, treatment, and rehabilitation, available on our website. Patients also received written brochures. A rehabilitation coordinator provided patients with all the necessary information and was available to them throughout the entire time.

Our study has several limitations. One is that it was not randomized. We had planned to conduct an ‘almost random’ approach. Originally, we intended that the first half of patients would be included in the control group, and the other half in the intervention group. But due to the COVID-19 pandemic, for some time we had to include patients simultaneously in the control or intervention groups based on their place of residence. Another limitation is a different place of residence, which may be associated with certain psychosocial characteristics which correlate to fatigue. Furthermore, the difference in distance from the hospital could influence the significant difference in fatigue between the two groups of patients (e.g., a more tiring drive to the hospital), so this must be considered when interpreting our results. Another limitation of the study is that targeted precision tests for the assessment of fatigue were not used, as we were also interested in other problems bothering patients. In addition, the number of included patients is still too small to enable a more detailed analysis of the connection between fatigue and other psychological factors. In addition, some patients decided to withdraw from the study and some patients did not respond to all parts of the questionnaires, therefore there are some missing data. Furthermore, different interventions were simultaneously implemented in order to achieve as much benefit for the patients as possible. Therefore, it was not possible to test the effect of a single intervention and to describe the contribution each intervention played in the treatment of fatigue. Due to waiting times for certain treatments in Slovenia, such as acupuncture or cognitive-behavioral therapy, some patients did not receive treatment immediately, or when they needed it most. But our study enables a realistic presentation of rehabilitation in our country and what possibilities exist for improving integrated rehabilitation. Finally, the number of patients included in the research is relatively small, but it represents 40% of all breast cancer patients detected annually in Slovenia, so we believe that the sample size is suitable for evaluating the effectiveness of non-integrated rehabilitation and integrative rehabilitation on fatigue in our country.

## Conclusions

Early individualized integrated rehabilitation is associated with a lower prevalence of chronic fatigue or fatigue interfering with usual activities in breast cancer patients in comparison to the control group of patients. A year after the beginning of treatment, patients from the intervention group reported significantly fewer problems also in the global health quality of life scale, pain, physical, emotional, cognitive, and social function scale in comparison with the control group.
